# Paracentesis Abdominalis1Read before the Pennsylvania State Veterinary Medical Association at Cresson Springs, September 3, 1895.

**Published:** 1895-10

**Authors:** James A. Waugh

**Affiliations:** Allegheny, Pa.


### PARACENTESIS ABDOMINALIS.[1]

BY JAMES A. WAUGH, V. S.,
ALLEGHENY, PA.

THE above-named operation is performed very frequently by modern veterinarians in this country, especially in the large cities and towns where they are called promptly in the early

[1] Read before the Pennsylvania State Veterinary Medical Association at Cresson Springs, September 3, 1895.

stages to relieve gastric and intestinal tympanitis, and it is usually attended by satisfactory results, though occasionally unfavorable symptoms supervened as a result of improper conditions.

It appears the importance of a correct performance of this operation has never been fully appreciated by a majority of the English-speaking veterinary profession, as it has generally been considered a very simple operation by educated and advanced veterinarians, while there is, what might properly be called, a conservative class of practitioners who deliberately postpone it until they call it their last resource; which is really almost no resource at all, when it is inconsistent to waste valuable time in deferring this operation until it is quite evident to all concerned that the poor animal is in a dying condition, and probably beyond human aid. However, it must not be surmised that I condemn common-sense or ordinary conservatism in either surgery or therapeutics, yet I cannot condone the ignorance and inability of those who are not able to perform urgent and necessary operations when called in the line of duty. We should not find fault with the general public, while it occasionally meets registered veterinary practitioners who are unable to perform any surgical operations, or even write a prescription, or their own names.

Veterinary text-books and some of our college professors afford us a rather brief and uninteresting description of this operation, and some even attempt to satisfy our student curiosity by declaring it is a French operation that appears very nice, but the patient generally dies from it. Remarks of that sort may prove serviceable in filling up space in a lecture, yet they are detrimental to confidence, and are of no value in general practice. It is not surprising that some of us have felt inclined and deemed it necessary to consult and study some of the standard text-books on the theory and practice of human medicine and surgery, but this is now no longer necessary, owing to the recent translations of excellent foreign works, and some very excellent additions from intelligent and well-educated English speaking veterinarians.

The old methods of performing this operation were rich in theory, but poverty-stricken in practice. However, I must admit my first operation was performed on one of "Uncle Sam's" mules, and it was very successful, although I had never received any clinical instruction, nor seen the operation demonstrated—not even on a cadaver. Nevertheless my next two cases resulted in incurable abscesses and great loss of condition, and it was finally deemed prudent to destroy the unfortunate patients, which in health had been worth six hundred dollars. I gave one of these mules five ounces tincture of aconite root *U S. P.*, without making it sick enough to attract any notice from the corral boss or mule-packers, who were frequently in the corral, and I afterward destroyed it with two drachms of sulphate of morphia *U. S. P.* This mule extended its hind limbs backward, front limbs forward, spread the limbs outward, resting the body on the abdomen and chin, and seemingly died without a struggle or any further suffering. I also killed the other mule in the same way. It is hardly necessary for me to mention that I realized this sort of practice would surely prove disastrous to financial or professional success in civil life, or even in the army.

In due course of time and with plenty of clinical material at my disposal for further experiments, and a firm determination to succeed, finally I adopted the following method of operation and treatment:

Instruments must be thoroughly cleaned, disinfected, and dressed with a preparation of carbolized olive oil—one part of carbolic acid to eight of olive oil—which keeps the instruments in proper condition for the operation, and the above mixture is also very serviceable in practice. I usually select a spot about five to eight inches in front of the antero-inferior spines of the ilium, on an imaginary line direct with the elbow, and with curved scissors carefully clip off the hair from spot about the size of a silver dollar; now rub on some carbolized olive oil, then use a thumb-lance or a dog seton-needle to make an incision through the skin and half way through the abdominal muscles, now push the trocar and canula through the external wound, directing it slightly forward and downward; grasp the canula firmly between the thumb and forefinger, and withdraw the trocar, then afterward complete the operation by pressing the other thumb and index finger against the sides of the wound while withdrawing the canula, and dress wound with carbolized oil; then put pad of marine lint over it, and secure with bandage or circingle.

I generally prefer to operate with the patient in a standing position with a trusty attendant at the head, but if lying down insist that a steady and fearless person hold the patient steady by placing knee on upper part of neck and both hands on bridle to prevent struggling and shifting position. We occasionally meet with desperate cases where no one is willing to trust himself at the head of the patient, and the operator is compelled to proceed without assistance. Twisting and bending of canulas convinced me that struggling and shifting position was a fruitful source of serious injury to the internal viscera and occasionally resulted fatally notwithstanding energetic treatment.

I have recently used Prof. Joseph Hughes' trocar and canula very successfully in tapping the stomach in several cases where I was unable to reach the gases by the ordinary trocar and canula. The stomach becomes greatly distended in some cases of acute indigestion, and there is really no trouble in reaching it with this instrument, which I cheerfully recommend and use. It is somewhat embarrassing to use the old-fashioned trocar and canula on an animal with an enlarged and tympanitic abdomen and fail to strike gas, and it is even liable to reflect discredit on the operator, but a proper use of the Hughes trocar and canula will prove the correctness of the diagnosis, and convert apparent defeat into laudable victory. A little study and experience will enable any veterinarian to become an expert and adept in the use of these instruments.

---

### BOOK NEWS.

THE book on *The Dog in Health and in Disease*, by Prof. Wesley Mills, of McGill University, published by Messrs. D. Appleton & Co., of New York, is just about to appear in its second edition. While the general plan has not been changed the work has been enlarged, several new illustrations added, and the whole carefully revised and brought up to date in every respect. The price will remain as before. The first edition met with a very warm reception, and this bespeaks for the second edition an equally well-recognized place among veterinarians as a text-book in colleges.

